# A Survey of Health Disparities, Social Determinants of Health, and Converging Morbidities in a County Jail: A Cultural-Ecological Assessment of Health Conditions in Jail Populations

**DOI:** 10.3390/ijerph15112500

**Published:** 2018-11-08

**Authors:** Robert T Trotter, Monica R Lininger, Ricky Camplain, Viacheslav Y Fofanov, Carolyn Camplain, Julie A Baldwin

**Affiliations:** 1 Department of Anthropology, Northern Arizona University, 575 East Pine Knoll Drive, Flagstaff, AZ 86001, USA; robert.trotter@nau.edu; 2 Department of Physical Therapy and Athletic Training, Northern Arizona University, 212 East Pine Knoll Drive, Flagstaff, AZ 86001, USA; monica.lininger@nau.edu; 3 Center for Health Equity Research, Northern Arizona University, 1395 S. Knoles Drive, Suite 140, Flagstaff, AZ 86011, USA; ricky.camplain@nau.edu (R.C.); carolyn.camplain@nau.edu (C.C.); 4 School of Informatics Computing and Cyber Systems, Northern Arizona University, 1296 S Knoles Drive, Suite Rm 304, Flagstaff, AZ 96001, USA; viacheslav.fofanov@nau.edu

**Keywords:** co-morbidities, jail populations, health disparities, incarceration and health, health policy

## Abstract

The environmental health status of jail populations in the United States constitutes a significant public health threat for prisoners and the general population. The ecology of jails creates a dynamic condition in relation to general population health due to the concentrated potential exposure to infectious diseases, difficult access to treatment for chronic health conditions, interruption in continuity of care for serious behavioral health conditions, as well as on-going issues for the prevention and treatment of substance abuse disorders. This paper reports on elements of a cross-sectional survey embedded in a parent project, “Health Disparities in Jail Populations.” The overall project includes a comprehensive secondary data analysis of the health status of county jail populations, along with primary data collection that includes a cross-sectional health and health care services survey of incarcerated individuals, coupled with collection of biological samples to investigate infectious disease characteristics of a county jail population. This paper reports on the primary results of the survey data collection that indicate that this is a population with complex and interacting co-morbidities, as well as significant health disparities compared to the general population.

## 1. Introduction

Jail populations in the United States experience significant public health threats that have serious implications for the broader community. The social and physical environment of jails creates a dynamic condition in relation to general population health due to the concentrated, often short term and repeated exposure to: (1) infectious diseases; (2) difficulty of access to treatment for chronic health conditions; (3) interruption in continuity of care for serious behavioral health conditions; as well as (4) on-going issues for the prevention and treatment of substance abuse disorders. While the impact of single conditions, such as HIV transmission, heart conditions, or severe mental illnesses (SMI) is critical for developing public health interventions and policies for jail systems, the multiplex impact of the convergence of multiple pandemics in jail systems is very poorly documented. This paper provides an empirical description of multiple interacting co-morbidities that impact the public health ecology of jails.

Approximately 12 million individuals cycle in and out of jails each year [1,2]. In addition, there are significant nationwide racial and ethnic disparities in the criminal justice system: 60% of jail and prison populations are ethnic and racial minorities, although they make up just 30% of the general US population [1,3]. Incarcerated minority populations are disproportionately burdened by higher rates of substance abuse and poor mental health, as well as chronic and communicable diseases [1,3].

Significant co-morbidity rates [4,5,6] create complex prevention and treatment conditions for both prisons and jails. Compared with the general population, incarcerated individuals have a higher burden of mental and neurological disorders, have high levels of stress, anxiety, sleep deprivation, and depression and have lower levels of self-efficacy as a result of the stigma and loss of social ties associated with being incarcerated [5,6,7,8,9]. Rates of many chronic diseases in incarcerated populations are more than double of those in the general population; examples include: diabetes (5.0% vs. 2.4%), chronic respiratory conditions (e.g., chronic obstructive pulmonary disease (COPD), 34.1% vs. 19.2%), and liver disease (10.0% vs. 0.6%) [1]. Similarly, rates of communicable diseases such as Hepatitis C, Human Immunodeficiency Virus (HIV), and tuberculosis [10] are higher in incarcerated populations (e.g., 3.5% vs. 0.4% for HIV among 25–34 year olds) [11]. Women [12], ethnic minorities [13], and older adults [14] are considered particularly at-risk for poor health outcomes. Further, people who do not have a permanent residence in between jail stays face greater risk of mortality due to treatable conditions [15].

Most of the public health research on incarcerated individuals to date is either focused exclusively on prisons or includes both prison and jail populations, even though the two populations encounter significantly different physical, social, and public health environments. Our project focuses on information specifically targeted at the more transient jail populations, given the probable higher public health impact of those populations on community level health disparities.

The average length of jail stay, nationally, is 25 days with an average national turnover rate of 55% per month, although smaller jails tend to have a higher turnover rate and shorter length of stay [16]. This relatively short term stay and rapid release from jail, compared to more stable prison populations, is a significant public health challenge for individuals and communities. Incarceration makes individuals more likely to relapse to substance abuse, and non-adherence to mental and physical health treatment programs [17,18,19]. Jail residents may also become an important vector for communicable disease being cycled in and out of jail populations. Continuity of care is severely impacted by cycling between jail and community and is associated with limited opportunity for stability in healthcare [20]. Medicaid provides a disproportionate number of incarcerated individuals with critical benefits prior to their incarceration, but those benefits are suspended or even eliminated upon incarceration [20,21]. This condition, in turn, causes a significant burden on local city and county budgets, since healthcare is a constitutional right for prisoners afforded by the Eighth and Fourteenth Amendments. (C.f. Estelle v. Gamble 429 US 97 (1976)) [22]. Consequently, the purpose of this work is to describe key characteristics of currently incarcerated individuals in a county detention facility, in relation to their health status, and public health impact on their community.

The primary research objectives addressed in this paper are as follows.

1. Describe Characteristics of Incarcerated Individuals in Northern Arizona. Describe the current county jail sample in terms of demographic information, income status, living conditions prior to incarceration, and general health measures.
2. Identify the Infectious Disease, Chronic Illness, Behavioral Health, Substance Use, and Global Health Conditions Prevalent in a County Jail. Of the current sample of incarcerated individuals in Northern Arizona, what is the prevalence of self-reported health conditions in the jail population, and what are the typical co-morbidity patterns identified by self-report?

## 2. Materials and Methods

The parent study of this current work, “Health Disparities in Jail Populations” [23] began (and continues) as a community engaged project following consultation with the local Criminal Justice Coordinating Council (CJCC). The county government is engaging in several “Collective Impact” [24,25] oriented projects targeted at improving the health and wellbeing of the community as a whole. Consultation with the CJCC council identified the county jail system as a significant public health priority that would benefit from an assessment utilizing a population health and cultural ecological framework in conjunction with policy development relating to the overall impact of the jail system on various aspects of the county healthcare delivery system. The county criminal justice system’s (Sherriff’s office, jail, courts, etc.) and the CJCC’s interest in conducting the project was fueled by both pragmatic experience and preliminary evidence that individuals in the population who experienced multiple incarcerations over time had a more noticeable impact on local health care services than the overall jail population. The detailed study protocols for the overall project are described in our protocol paper [23]. The purpose of this paper is to provide a baseline description of key characteristics of currently incarcerated individuals in a Northern Arizona county detention facility, with special emphasis on self-reports of global health status, utilization of health services before incarceration, and recognition of the complex interaction of 28 specific health conditions including chronic illness, infectious disease, mental health conditions, and substance use. We feel that this basic descriptive information sets an important framework for more targeted assessments of the health disparities that impact this fluid population as well as their public health impact on the community. We believe the results reported below have important implications for existing public health programs targeted at incarcerated populations, as well as community and county level health policy that addresses local, rather than generic national level conditions. 

### 2.1. Instrument Development

The project jail-based survey includes specific items about (1) demographic information, (2) social determinants of health, (3) respondents’ experience with the criminal justice system, (4) healthcare service utilization patterns, (5) self-reported experience of communicable disease, chronic illness, substance abuse, and behavioral health issues, and (6) health behaviors (e.g., physical activity and smoking) of respondents. The survey instrument is comprised of 158 items across 15 scales adapted from existing national health surveys of general populations [26], other measures of relevant health and well-being constructs with high previously-demonstrated validity and reliability [27], as well as instruments targeted at assessing incarcerated populations [28].The final instrument includes questions relating to a broad list of health domains; specifically, a range of communicable diseases, commonly-occurring chronic conditions, issues related to behavioral health and well-being, and other related constructs (e.g., global self-rated health status), as well as a comprehensive set of questions assessing demographic and socioeconomic characteristics. The majority of questions were taken from: (1) the National Health Interview Survey (NHIS) [29]; (2) the National Health and Nutrition Examination Survey (NHANES) [26]; (3) the Behavioral Risk Factor Surveillance System (BRFSS) [30]; (4) Behavioral Risk Factor Surveillance System (BRFSS ACE module) [31]; (5) Patient Health Questionnaire (PHQ) [32]; (6) Patient-Reported Outcomes Measurement Information System (PROMIS) [33]; (7) National Survey of Midlife Development in the United States (MIDUS) [34]; (8) Brief Symptom Inventory (BSI) [35]; (9) Cohen’s Perceived Stress Scale (PSS) [36]; (10) International Physical Activity Questionnaire (IPAQ) [37]; and (11) the National Inmate Survey [38]. 

Our initial instrument was assessed through a pilot cognitive debriefing process [39,40], to ensure: (1) a consistent understanding of questions across participants and (2) an alignment of participants’ understanding of items with the original intent of the questions. Survey items that were problematic tended to fall into three categories: (1) uncertainty about definitions of items or recognition of some diseases; (2) a request that additional questions be added; and (3) the desire for additional response options (e.g., add “internet” as a response option to the question querying the type of place one goes to for healthcare). We were able to accommodate concerns of type 1 and 2 and subsequently constructed the final version of the instrument by imbedding the definitions that were provided to interviewers (e.g., in the National Health and Nutrition Examination Survey (NHANES)) within the questionnaire, and adapting items and item responses where necessary. The final instrument face validity and individual item comprehension were assessed through a pilot test and debriefing process with incarcerated individuals in the county jail. We wanted to determine if there were any significant problems with reading level, item comprehension, with sensitivity of questions (especially alcohol and drug questions), and the range of time it took to complete the survey (between 25 and 50 min for the sample). The pilot test indicated that even the slowest reader could complete the survey in the allotted time. Reading comprehension was generally acceptable; a few participants requested further clarification about three of the diseases listed (COPD, Gout, Angina) and these queries were addressed by additional definitions and individual interactions with respondents. The alcohol and drug questions were not considered sensitive, according to respondents. 

### 2.2. Subject Recruitment

The overall ecology of jail provides an important physical and socio-cultural framing for the public health findings for our study. Our survey respondents were incarcerated in a local county jail. There are approximately 10,000 individual incarcerations recorded in the jail, annually. The jail is structured around 4 pods with a total of 22 dorms embedded in the pods. Each dorm typically has a common area with multiple tables, chairs, a television set, a bank of phones, and a common toilet and shower facility. The lower security pods (minimum security, work release, and trustee dorms) are primarily large open dorm facilities with beds arranged around the commons space. The higher security pods consist of a commons area, with multiple 2–4 bed rooms connected through doors to the main room. Each pod has a “program room” where the interviews were conducted and various religious, treatment, and social programs are conducted each week. Each dorm has associated sanitary facilities and common space for social interaction. A total of 598 beds are available in the facility, with 480 beds occupied being identified as full capacity, given constraints on security and administrative segregation.

Survey participants were recruited based on a stratified purposive sampling strategy [41,42,43]. Our target sample was between 12 and 15 individuals per dorm to provide a representative sample of all three primary segmentation elements (male/female, security designation, and race/ethnicity). We established a total recruitment target of 200 individuals (approximately 40 percent of the total available beds excluding the restricted dorms), and approximately 54 percent of the average bed occupancy for the facility. That sample size allowed us to achieve a representative non-probabilistic sample of the overall jail population, based on the general demographics reported by the jail. Four “restricted” dormitories were excluded from the sample design: (1) a dorm housing juveniles being charged as adults; (2) an “administrative confinement” dorm (lockdown); (3) a dorm for individuals diagnosed with SMI (severe mental illness) and not considered competent to consent to participation; and (4) an administration dorm that houses protected individuals, such as former officers.

The inclusion criteria for the survey consists of (1) being incarcerated in the County Detention Center at the time of the survey, (2) being 18 years old or older, (3) being able to read English, and (4) providing informed consent for participation. We excluded individuals if they (1) resided in a restricted dormitory in the Jail, (2) decided to not provide informed consent for participation, and/or (3) were considered unable to consent due to a cognitive impairment. There was no exclusion on the basis of sex, ethnicity, or health status. Due to high comorbidity of SMI and substance abuse in jail populations [42], we anticipated enrolling these individuals if they were a resident of the 18 dorms and able to complete the informed consent process. Finally, although pregnant women may be a part of the jail population, they were neither specifically targeted nor were they excluded. 

Participants were recruited in each individual dorm at approximately 8 a.m. (after breakfast) on Mondays and Wednesdays and invited to participate in an interview session either that day or within three days of recruitment. A study team member and a jail staff member described the overall project to the dorm residents, including the basic informed consent information, the purpose of the study, and the incentive for the study. Interested participants who met eligibility requirements signed up on a general recruitment sheet, and were consequently assigned to a program time slot to complete the survey. At the appointed time, County Detention personnel escorted residents, in groups of five, to a nearby (pod specific) program room equipped with a one-way mirror, video recording, chairs and tables, and materials used for programs. Individuals had the opportunity to decline participation at this point in the process, by deciding not to accompany the officer. If any of the scheduled individuals were not available at the time of escort, listed alternates were allowed to be included in the process. Upon entry into the program room, a study team member reviewed the purpose of the study, reiterated all of the elements of the informed consent process, had individuals read the informed consent document, and requested signed informed consent to proceed. Three individuals declined participation after listening to the informed consent process. If, at any time after an individual had signed the informed consent form and subsequently decided to halt participation, they were allowed to do so with no consequences. However, we did not have any individuals request this option either during or after the survey.

Ethics: Our study involves a vulnerable population—individuals incarcerated in jail. Persons with SMI’s, homeless individuals, and pregnant women were not specifically targeted, but were not excluded if they were resident in one of the 18 sample dorms. The IRB of record is the Northern Arizona University IRB (Approval #1067490). All investigators were required to complete all CITI modules relating to vulnerable populations, incarcerated individuals, as well as the other required CITI modules required by the university. Investigators were required to go through an approved jail training program for volunteers, including PREA training [43] and safety training, and were required to follow all procedures and regulations within the County Detention Facility. Additionally, investigators were under an obligation or a “duty to inform” when an incarcerated individual posed a threat to either themselves or others (and this was discussed in the informed consent procedure). Finally, to preserve confidentiality, audio monitoring and recording were suspended during survey sessions. However, there were special security considerations that included video monitoring and the presence of jail personnel outside of the “program room” who could be contacted in an emergency.

The confidentiality of the data is maintained through the use of a secure CADI system (iPAD computer assisted data collection) and secure data transfer and storage of all research information on dedicated encrypted servers, as well as by limiting access to all data to key research personnel only. No individuals will be identified or identifiable in reports or publications. 

The consent form included three sections: (1) an explanation of the survey; (2) explanation of the collection of biological samples; and (3) permission to access jail incarceration and medical records. If an individual did not consent to the study survey, they were escorted back to their pod. Participants were allowed to continue if they did not consent to collection of biological samples and/or access to jail medical records. Following the consent process, the individuals were given a second generation iPad to complete the survey using the Qualtrics^TM^ (Qualtrics, Provo, UT, U.S.) Office Survey Application, a platform for administering surveys without an internet connection. Participants were provided with a $15 incentive in the form of commissary privileges during incarceration or a gift card that was sent to the participant after release from jail. For individuals transferred to the Department of Corrections (prison), the incentive was forwarded to their destination facilities.

### 2.3. Measures

Participants self-reported sex (male or female), race (American Indian or Alaska Native, white, or other), ethnicity (Hispanic/Latino or non-Hispanic/Latino), education level (less than a high school diploma or GED (General Equivalency Diploma), high school diploma or GED, or some college or higher), marital status (divorced or widowed, married, separated, or single), and annual household income. In addition to demographic information, we collected data on participants’ social environment. Participants self-reported living status prior to incarceration, whether they had ever been homeless, employment status prior to incarceration, and health insurance status. Participants also reported time since most recent health care visit and the number of emergency room visits they had 12 months prior to incarceration.

General health was self-reported as excellent, very good, good, fair, or poor. Height and weight were self-reported. Body mass index (BMI) was calculated as weight (in kilograms (kg)) divided by height in meters, squared. BMI was categorized as normal (<25 kg/m^2^), overweight (25–30 kg/m^2^), and obese (>30 kg/m^2^). Participants reported whether a doctor or health professional had ever told them they had arthritis, bronchitis, a liver condition, asthma, hypertension, high cholesterol, diabetes, or prediabetes. Participants also self-reported mental health conditions including attention deficit disorder (ADD) or attention deficit hyperactivity disorder (ADHD), anxiety, bipolar disorder, depression, schizophrenia, or post-traumatic stress disorder (PTSD). Participants indicated if they had ever been diagnosed with Hepatitis B, Hepatitis C, Tuberculosis, or HIV.

Participants also indicated if they had ever used heroin, other opiates, methamphetamine, other amphetamines, methaqualone, barbiturates, tranquilizers, crack, cocaine, PCP, ecstasy, LSD, or marijuana. They also self-reported alcohol use in the 30 days prior to incarceration.

### 2.4. Statistical Analysis

**Figure 1:**
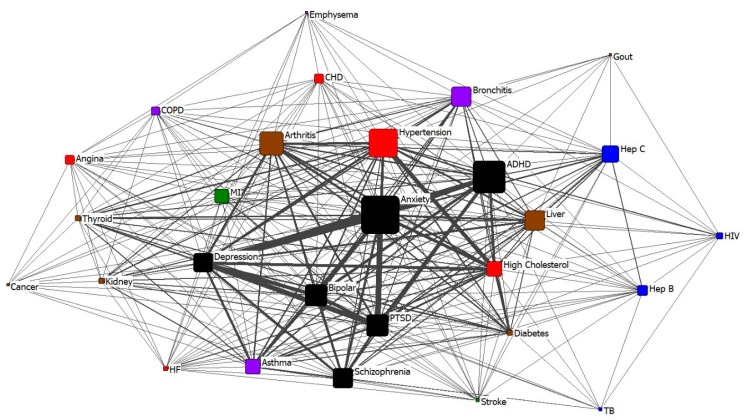
Network Relationships between Self-Reported Health Conditions in Jail Sample. Morbidity Type Legend. 1= mental health; black. 2 = infectious disease; blue. 3 = organs and cancer; brown. 4 = lungs/pulmonary; purple. 5 = acute cardiac; green. 6 = heart and vascular; red.

The primary research questions were answered through measures of central tendency (means and standard deviations for continuous data, medians and range for skewed data) and frequencies with percentages for categorical variables. All analyses were performed using SPSS (version 24; IBM Corp, Armonk, NY, USA). The network analysis presented below was conducted by importing the survey co-morbidity matrix into UCINET 6 (Analytic Technologies, Lexington KY, USA), constructing the two centrality measures, and exporting the UCINET file into NETDRAW [44] to construct Figure 1 (visual representation of the co-morbidity inter-connections).

## 3. Results

Following standard data integrity and data cleaning protocols, our final sample size was 199 adults (78.9% male, 21.1% female). The majority of the sample were single (53.7%), American Indian/Alaskan Native (58.8%), and male (78.9%). In comparison, the average jail census in the county is 80–84% male, 16–20% female, 52% American Indian or Alaska Native, 32% non-Hispanic white, and 13% Hispanic. In general, participants either had a household income of less than $10,000 annually (45.9%) or did not know their annual income (17.0%). It took participants 41.1 ± 12.6 min to complete the questionnaire. The median length of stay (incarceration) in our sample was 46 days (range 2–898 days). The survey participants were approximately evenly divided between those having less than a high school education, those with a high school diploma or GED and those with some college or higher (Table 1).

Regarding the social environment, 70.1% of participants lived in a house, apartment, or mobile home prior to incarceration and 42.4% indicated they had ever been homeless (Table 2). Additionally, 45.7% of participants worked for pay prior to incarceration. Prior to incarceration, 79.2% had health insurance, 43.9% had not seen a healthcare provider within 6 months, and 69.6% had been to an emergency room within 12 months (Table 2).

Most individuals considered themselves to be in ‘good’ (34.9%) or ‘fair’ (29.2%) general health. (Table 3). Additionally, 61.3% of participants were overweight or obese. Of the medical conditions participants self-reported, hypertension (35.9%), high cholesterol (17.8%), and arthritis (17.5%) were reported the most followed by asthma (14.9%), diabetes and prediabetes (12.3%), liver conditions (11.9%), and bronchitis (7.2%). Although not as common, at least two participants had each of the following self-reported medical conditions: gout, congestive heart failure, coronary heart disease, angina (angina pectoris), heart attack (myocardial infarction), stroke, emphysema, thyroid condition, chronic obstructive pulmonary disease, weak or failing kidneys, or cancer as further discussed in Table 4. Of the mental health conditions participants self-reported, anxiety a (36.5%), and depression (33.5%), and PTSD (26.3%) were reported the most followed by ADHD (23.1%), bipolar disorder (19.9%), and schizophrenia (11.2%). For infectious diseases, Hepatitis B and C were the most commonly reported (2.5% and 7.0%, respectively). HIV infection was reported by five individuals of 102 who were tested (2.5% of the entire sample). Over 80% of participants reported ever using marijuana, while 57.7% reported ever using methamphetamine and 49.5% reported ever using cocaine (Table 3). In the 30 days prior to incarceration, 76.1% reported consuming alcohol.

### Comorbidity Conditions

The survey presented 28 health conditions that were singly or in combination identified as important conditions effecting incarcerated individuals, based on a review of the literature on incarceration and health. Each respondent was asked to self-identify all of the health conditions that they had been informed (by a health care professional) they had. We subsequently approached these questions from a multiple response perspective (i.e., for each individual we indicated all of the conditions reported, rather than simply summing individual conditions). Table 4 identifies the dyadic relationships among the reported health conditions, excluding alcohol and drug use. For example, reading down the arthritis column, arthritis was paired with gout by one respondent, while arthritis was paired with asthma by 10 individuals. The table supports the basic hypothesis that health and health care models for incarcerated individuals is an ecologically complicated system of interconnected morbidities that need to be systematically addressed.

Another way of describing these converging morbidities is to represent all of the interconnections between the 28 identified co-morbid health conditions through the lens of a network diagram. In Figure 1, each health condition is represented by a node in the network diagram. The connections between them (identified co-morbidity matches from the matrix) are represented by the lines between the nodes. The thickness of the lines between nodes represents the number of times the dyadic co-morbidity is identified by multiple individual respondents. The size of the node represents the number of times that the condition is listed as a comorbidity.

A visual inspection of the nodes indicates that co-morbidities tend to cluster around “central” nodes. One potential use for this data would be to look at the strongest clusters (i.e., ego-centric models), and to determine the most common comorbidity clusters. That would allow the health care system to do a quick check for the most common comorbidities in the cluster, without having to screen for every possible co-morbidity. Later analyses will include both alcohol and drug use as co-morbidities, however we found that the inclusion of those conditions for this paper visually obscured the interconnections between all other health conditions, and diminished the focus on all other co-morbidities.

One type of ecological measure that appears to be useful in the assessment of the self-reported co-morbidities is “network centrality”. Table 5 identifies the “degree centrality” and the “betweeness centrality” of each reported condition. Degree centrality is a simple measure of the number of connections (ties) that a particular node has with all other nodes in the network [45,46]. The higher the number of ties, the more a node acts as a central connection point in a matrix of conditions. The 12 highest degree centrality measures are identified in Table 5. In line with our hypothesis relating to the need to identify key clusters of conditions, the top centrality measures indicate the overall number of connections between chronic health conditions infectious disease, and behavioral health conditions. These conditions are also accompanied by high “betweenness centrality” [47,48]. This centrality measure references the condition that a node has a higher or lower centrality based on the fraction of shortest paths between all node pairs that pass through the node of interest. Betweenness can be interpreted as a measure of the “influence” a node has over the spread of information (in this case the information is the connection between co-morbidities―i.e., it acts as a “go-between” condition that links sub-clusters of morbidities).

Each of these measures, both individually and in aggregate, support our contentions about the complexity of the comorbidity environment, and support a “syndemics” oriented approach to public health in jails [49]. As a consequence, each of the high centrality health conditions in Table 5 should be identified as an important point for investigating the convergence of various sets of co-morbidities, and should support the formulation of “collective impact” friendly policy supporting public health programs in jails.

## 4. Discussion

This exploratory pilot study of converging health conditions and the sociocultural ecology of county prisons produced a very rich data set designed to both inform our community partners (the criminal justice coordinating council) and to provide a framework for additional assessment of the public health impact of incarcerated populations. The survey results demonstrate that jails present a very complex public health environment. This paper provides locally actionable baseline data on a significant number of previously identified health conditions reported in jail populations. Slightly more than 60 percent of the respondents self-identified at least one of the targeted health conditions in the survey, and all 28 conditions were reported by at least two individuals surveyed. Most of these conditions have been individually assessed, or assessed in small clusters of related disease categories, such as substance abuse, or chronic illnesses, or sexually transmitted disease. In line with those studies, our population has a higher prevalence of mental and physical health conditions compared with the general population of Northern Arizona [50]. For example, our study population reported lower self-reported positive general health (excellent, very good, or good) compared to the general population of Coconino County [50] (63% vs. 87%, respectively). Additionally, a higher percentage of individuals incarcerated in jail reported high blood pressure (36%) compared to the general population of Coconino County (19%) [50]. Since that indicator correlates with projected five year mortality rates, this finding has important projective global health and community health policy implications [51].

There were some limitations to this study which should be noted. First, medical information was self-reported by jail residents. In future analyses, we will have access to electronic medical records provided by the jail. Using these records, we will validate these self-reported findings. Another limitation includes selection bias. The median length of stay in our sample was 46 days (range 2–898 days) compared to 2 days among the full jail population (data not shown), and compared to 25 days nationally. Our sample of individuals who reside in jail for longer lengths of time includes individuals who are unable to make bail, are serving sentences, or awaiting trial, and consequently policies to improve healthcare in jail facilities may benefit this population more than those with shorter lengths of stay.

Additionally, our stratified purposive sample of individuals from one semi-rural, county jail may not be representative of individuals incarcerated in jail in the United States, as a whole. Thus, some of our findings may not be generalizable to other facility, state, or national populations. Also, due to the unique make-up of our study population, comparisons of disease prevalence to the general population may not be appropriate in some circumstances, and the overall configuration of comorbidities may differ by group and region.

There were also many strengths of this study. Unlike most of the other studies, we assessed the complex interactions of the targeted health conditions both within individuals, and in the population as a whole. It is very clear from the data that co-morbidities in an incarcerated population present a special challenge to detention centers and to incarcerated individuals in terms of treatment priorities, health policy, and general population health characteristics. Since federal policy prohibits the use of Medicare/Medicaid reimbursement to treat prisoners, jails in particular must have policy and procedures in place to deal with both acute and chronic illnesses. Federal, state, and county policies need to allocate more funds and resources to the constitutional requirement [22] of healthcare in correctional facilities.

Policies that train detention officers in more than basic first aid are warranted to combat the lack of knowledge of chronic and acute illnesses as well as the widespread ignorance and stigma of mental health. Because jail personnel are often the first to respond to potential medical situations, they should be able to detect early signs of illness and injury as well as identify and respond to life-threatening situations. To improve mental health literacy among jail staff, mental health first aid may improve healthcare in jail facilities. The standard requirements in all jail facilities should be uniform and potentially overseen by a governing body.

Existing healthcare policies and procedures in the jail may create structural and perceived barriers. For example, even though an acute illnesses may be treated in a single visit, institutional barriers, such as a $10.00 charge for a single visit to the nurse, may still prevent an individual from getting treatment. For the population we surveyed, healthcare visit costs were a common barrier to seeking treatment unless the individual perceived the problem as particularly severe. Similarly, chronic conditions may warrant multiple visits to medical and often result in medication delays due to differences in formularies or a need to confirm the condition. This is especially true for SMIs, but also true for diabetic conditions, heart conditions, or HIV. Overall, individual conditions present serious logistical problems for detention facilities, while multiple conditions are more difficult to address.

A quick inspection of the comorbidity diagram above indicates that there is a serious environmental barrier to addressing the public health impact of incarceration both within the jail system, and when the incarcerated individuals return to their communities. Dealing with all of the conditions for this population, given the average time of incarceration for each individual, creates serious operational and policy issues that need to be addressed on a system wide basis, rather than by detention facilities alone. The overall public health burden in this population is much heavier than that of the general county population, and confirms a need for a multi-sectorial approach to public health issues within the jail and, after incarceration, within the community.

## 5. Conclusions

Please summarize this research with a few sentences or you could remove some paragraph in the discussion section to this section. We recommend addressing the primary social and environmental impacts on the health of incarcerated individuals by taking the position that a simple categorical approach (single disease, single solution) would ultimately fail to appropriately identify the public health ecology of incarceration and its impact on population health [52]. One of our working assumptions is that neither the jails, nor external community programs are appropriate venues to conduct all prevention and intervention programs in all four areas of the converging morbidities in our study (chronic illness, behavioral health, infectious disease, and substance abuse). Rather, some form of integrated multi-sectoral approach is needed to comprehensively address public health needs of this population. At the same time, jails can and should play a pivotal role in addressing the overall impact of incarceration on public health. Thus, we believe a multi-sectoral or collective impact framework [24] will be necessary to reduce the “broken public health” system in jails. Our long term goal, based on the need to identify and explore the complex relationship among these intersecting conditions, is to produce a model for prevention and intervention programs in all four areas of health. This could potentially result in significant cost reduction for healthcare among incarcerated individuals and jail facilities. In the future, we will be assessing the accuracy of self-reported conditions in comparison to the actual health records of our respondents, and analyzing the interaction of health conditions with incarceration data, social determinants of health information, as well as previous childhood trauma and issues surrounding access to health care services. Future analyses from this data set will also include more detailed analyses of specific conditions and their severity, disease prevention through health behaviors such as physical activity, and the associations between the two.

## Figures and Tables

**Table 1:** Descriptive Statistics of Demographic Information.

Variables	Frequency	Percent
Sex (*n* = 199)		
Male	157	78.9
Female	42	21.1
Education (*n* = 199)		
Less than high school	60	30.2
High school diploma or GED	76	38.2
Some college or higher	63	31.6
Race (*n* = 199)		
American Indian/Alaska Native	117	58.8
White	55	27.6
Other (Black, Asian, and Other)	31	15.6
Ethnicity (*n* = 196)		
Hispanic/Latino	29	14.8
Not Hispanic/Latino	167	85.2
Marital Status (*n* = 192)		
Divorced or Widowed	43	22.4
Married	31	16.1
Separated	15	7.8
Single	103	53.7
Annual Household Income (*n* = 194)		
0–9999	89	45.9
10,000–19,999	19	9.8
20,000–29,999	14	7.2
30,000–39,999	13	6.7
40,000–49,999	6	3.1
Greater than or equal to 50,000	20	10.3
Don’t know	33	17.0

**Table 2:** Descriptive Statistics of Social Environment.

Variables	Frequency	Percent
Living Status Prior to Incarceration (*n* = 194)		
House, apartment, or mobile home	136	70.1
On the street or homeless shelter	39	20.1
Other	19	9.8
Ever Been Homeless (*n* = 163)		
Yes	69	42.4
No	93	57.6
Employment Prior to Incarceration (*n* = 199)		
Working for pay	91	45.7
Self-employed	30	15.1
Looking for work	39	19.6
Permanently Disabled	12	6.0
Student	7	3.5
Other	20	10.1
Health Insurance Status (*n* = 197)		
Insured	156	79.2
Uninsured	41	20.8
Time Since Most Recent Health Care Visit (*n* = 189)		
≤ 6 months	106	56.1
6 months–≤ 1 year	36	19.1
1–2 years	25	13.2
≥2 years	22	11.6
Number of Emergency Room Visits * (*n* = 199)		
None	60	31.4
1	53	26.9
2–3	56	28.3
≥4	26	13.3

* In the past 12 months.

**Table 3:** Descriptive Statistics of Self-Reported Health.

Variables	Frequency	Percent
General Health (*n* = 195)		
Excellent	16	8.2
Very good	39	20.0
Good	68	34.9
Fair	57	29.2
Poor	15	7.7
Body Mass Index Categories (*n* =1 91)		
Underweight or Normal (<25 kg/m^2^)	74	38.7
Overweight (25–29.9 kg/m^2^)	72	37.7
Obese (≥30 kg/m^2^)	45	23.6
Medical Conditions		
Hypertension (*n* = 192)	69	35.9
High Cholesterol (*n* = 191)	34	17.8
Arthritis (*n* = 194)	34	17.5
Asthma (*n* = 195)	29	14.9
Prediabetes or Diabetes (*n* = 196)	24	12.3
Liver Condition (*n* = 193)	23	11.9
Bronchitis (*n* = 195)	14	7.2
Mental Health Conditions		
Anxiety (*n* = 197)	72	36.5
Depression (*n* = 197)	66	33.5
PTSD (*n* = 198)	52	26.3
ADD/ADHD (*n* = 195)	45	23.1
Bipolar Disorder (*n* = 196)	39	19.9
Schizophrenia (*n* = 196)	22	11.2
Infectious Diseases		
Hepatitis C (*n* = 199)	14	7.0
HIV (*n* = 102) *	5	4.9
Hepatitis B (*n* = 199) *	5	2.5
Tuberculosis (*n* = 199) *	4	2.0
Substance Ever Use		
Marijuana (*n* = 195)	159	81.5
Cocaine (*n* = 196)	97	49.5
Methamphetamine (*n* = 196)	113	57.7
Other Amphetamines (*n* = 195)	64	32.8
LSD (*n* = 194)	61	31.4
Other Opiates (*n* = 195)	59	30.3
Heroin (*n* = 196)	57	29.1
Crack (*n* = 193)	51	26.4
Ecstasy (*n* = 193)	50	25.9
Barbiturates (*n* = 195)	46	23.6
Tranquilizers (*n* = 196)	39	19.9
PCP (*n* = 194)	24	12.4
Methaqualone (*n* = 195)	18	9.2
Alcohol Use ^a^ (*n* = 188)		
Yes	143	76.1
No	45	23.9

Abbreviations: ADD: Attention Deficit Disorder; ADHD: Attention Deficit Hyperactivity Disorder; PTSD: Post Traumatic Stress Disorder; HIV: Human Immunodeficiency Virus; PCP: Phencyclidine; LSD: Lysergic acid diethylamide. ^a^ In the previous 30 d prior to incarceration. * indicates a cell size < 11.

**Table 4:** Co-Morbidity Matrix Showing Dyadic Relationships with Self-Reported Health Conditions for incarcerated individuals.

	Arthritis	Gout	HF	CHD	Angina	MI	Stroke	Emphysema	Thyroid	Bronchitis	Liver	COPD	Asthma	Kidney	Cancer	Hypertension	High Cholesterol	Diabetes	Hep B	Hep C	TB	HIV	Anxiety	Depression	Bipolar	Schizophrenia	PTSD	ADHD
Arthritis																												
Gout	1																											
HF	1	0																										
CHD	2	0	2																									
Angina	1	0	1	1																								
MI	3	0	3	3	3																							
Stroke	1	0	2	1	2	2																						
Emphysema	1	0	0	1	1	1	0																					
Thyroid	6	0	2	1	2	2	0	0																				
Bronchitis	7	2	0	1	1	1	0	1	3																			
Liver	7	1	2	1	1	1	1	0	4	4																		
COPD	3	0	0	1	1	1	0	1	1	2	1																	
Asthma	10	0	2	0	1	2	1	0	4	5	3	2																
Kidney	4	0	2	1	1	1	0	0	2	2	2	1	4															
Cancer	2	0	0	0	1	1	0	0	1	0	0	1	2	1														
Hypertension	18	1	3	2	2	4	2	1	8	5	12	0	9	4	1													
High Cholesterol	15	0	4	2	0	3	2	1	4	6	4	3	8	4	0	29												
Diabetes	8	0	2	1	0	1	2	0	4	5	5	1	6	1	0	9	19											
Hep B	1	0	1	1	0	1	1	0	0	1	2	0	1	0	0	1	2	1										
Hep C	4	1	0	2	0	2	1	1	0	2	8	1	3	0	0	3	7	2	4									
TB	0	0	0	0	0	0	0	0	0	0	0	0	1	0	0	0	2	0	1	0								
HIV	0	1	0	0	0	0	0	0	0	1	1	0	0	0	0	4	0	0	1	1	1							
Anxiety	17	1	2	1	1	3	2	1	9	11	14	3	19	6	3	17	25	6	2	8	3	1						
Depression	18	0	2	1	1	3	2	1	8	9	12	3	16	6	3	18	24	8	0	7	2	0	58					
Bipolar	10	0	3	1	2	3	1	0	7	5	5	1	10	4	2	8	11	2	1	4	2	1	34	31				
Schizophrenia	7	0	2	0	1	2	1	0	3	2	5	1	8	3	1	5	7	2	1	2	2	1	19	19	17			
PTSD	14	0	3	2	2	4	2	0	7	5	8	2	15	5	2	18	12	3	1	5	3	1	46	42	28	19		
ADHD	9	2	3	2	1	3	2	1	5	6	7	1	8	3	0	9	17	3	1	7	1	1	34	30	22	11	26	

Abbreviations: Angina: Angina Pectoris; MI: Myocardial Infarction; Thyroid: Thyroid Condition; Liver: Liver Condition; COPD: Chronic Obstructive Pulmonary Disease; Kidney: Weak or Failing Kidneys; Hep B: Hepatitis B; Hep C: Hepatitis C; TB: Tuberculosis; HIV: Human Immunodeficiency Virus; PTSD: Post Traumatic Stress Disorder; ADHD: Attention Deficit Hyperactivity Disorder.

**Table 5:** Centrality Measures for Comorbidity Conditions.

Condition	Degree Centrality	Betweeness Centrality
Anxiety	346	10.351478
ADHD	215	8.6119423
Hypertension	193	7.442946
Arthritis	170	6.1652584
Bipolar	215	5.5080433
PTSD	275	5.5080433
Schizophrenia	141	5.0631657
Liver	111	5.0189047
Depression	324	4.7398615
Hep C	75	4.1134052
High Cholesterol	211	3.7165437
Asthma	140	3.5543275
